# Polymer Optical Fiber Tip Mass Production Etch Mechanism to Achieve CPC Shape for Improved Biosensor Performance

**DOI:** 10.3390/s19020285

**Published:** 2019-01-12

**Authors:** Hafeez Ul Hassan, Ole Bang, Jakob Janting

**Affiliations:** 1 DTU Fotonik, Department of Photonics Engineering, Technical University of Denmark, Frederiksborgvej 399, 4000 Roskilde, Denmark; hafha@fotonik.dtu.dk; 2 DTU Fotonik, Department of Photonics Engineering, Technical University of Denmark, Ørsteds Plads, 2800 Kgs. Lyngby, Denmark; oban@fotonik.dtu.dk

**Keywords:** fiber optic sensors, polymers, blood or tissue constituent monitoring, etching, nonimaging optics

## Abstract

We report on a simple chemical etching method that enables nonlinear tapering of Polymer Optical Fiber (POF) tips to manufacture Compound Parabolic Concentrator (CPC) fiber tips. We show that, counter-intuitively, nonlinear tapering can be achieved by first etching the core and not the cladding. The etching mechanism is modelled and etched tips are characterized both geometrically and optically in a fluorescence glucose sensor chemistry. A Zemax model of the CPC tipped sensor predicts an optimal improvement in light capturing efficiency of a factor of 3.96 compared to the conventional sensor with a plane-cut fiber tip. A batch of eight CPC fiber tips has been manufactured by the chemical etching method. The batch average showed an increase of a factor of 3.16, which is only 20% less than the predicted value. The method is reproducible and can be up-scaled for mass production.

## 1. Introduction

Optical fiber sensors based on Polymer Optical Fibers (POFs) have a number of advantages compared to sensors based on silica fibers, such as being flexible, durable, biocompatible, and biodegradable [1,2]. These features make them particularly suitable for biosensors [3,4,5,6,7,8] and chemical sensors [9,10,11,12]. Among these sensors, the glucose sensor is of particular interest as it can be used for optimal control of diabetes [13,14,15]. Different techniques can be employed for glucose sensing, the most important of which is fluorescence measurement. A POF-based fluorescence glucose sensor has already been developed by Medtronic [16]. One important limitation to such fluorescence sensors is the weak fluorescence signal, which can compromise the sensor performance [17]. This limitation can be overcome by increasing the numerical aperture of the fiber in order to collect more fluorescence. Although linear tapering of the fiber tip can increase the fiber numerical aperture, the maximum pickup efficiency is achieved using a nonlinear shape known as a Compound Parabolic Concentrator (CPC), as demonstrated in our previous work [16,18]. The CPC is a well-known profile that has been demonstrated for optimal light pickup efficiency [19] in various applications, such as increasing the light capture efficiency in solar energy systems [20] and improving the coupling of LED light into fibers [21].

In our previous work on POF-based glucose sensors, we manufactured POF Compound Parabolic Concentrator (POFCPC) tips using two different methods: (1) Heat-and-pull [4,16] and (2) Femtosecond (fs) laser micromachining [18]. The methods were characterized in terms of their improvement in light capturing efficiency compared to the conventional sensor with a plane-cut fiber tip, which Zemax modelling of an ideal CPC tip predicts to be a factor of *η* = 3.96 [16,18]. In the heat-and-pull method, a local area of the fiber is heated and pulled to parabolically taper it. The tapered region is then cut in the middle with a scalpel and polished, resulting in two CPC fiber tips. Although it is relatively easy to manufacture CPC fiber tips using this method, it requires stringent control of several experimental parameters, such as heating time, pulling speed, etc. This limits the reproducibility of the CPC fiber tips on top of the fact that the method is not scalable and does not allow for mass fabrication. Also, this method does not give an ideal CPC shape and therefore one cannot achieve the maximum increase in the fluorescence pickup efficiency, but only a factor of *η* = 1.7 [16]. In contrast, fs laser micromachining provides a more accurate CPC shape and the improvement factor for the fluorescence pickup efficiency using this technique is *η* = 3.5, which is close to the ideal value, *η* = 3.96, predicted by Zemax modelling [16]. However, fs laser micromachining is a complex process giving only one CPC at a time, and the use of a fs laser and position control leads to a very high cost. Considering all these factors, both heat-and-pull and fs laser micromachining are not industrially viable and suitable for batch production of CPC fiber tips. Therefore, an alternative method is required, which should be simple, cost effective, and scalable to CPC fiber tip batch manufacturing. One particular method that has such potential is chemical etching.

In silica glass fibers, chemical etching has been used to make linear [22,23,24,25,26] and nonlinear (e.g., parabolic and hemispherical) fiber tip shapes [27,28]. In all cases different hydrofluoric acid (HF) etch rates for core and cladding contribute to the tip shaping. Other studied, more or less time dependent, tip shaping parameters are: The meniscus between the HF solution and a top layer of oil [22], etchant composition [23], meniscus around fiber at interface between HF etchant and organic solvent together with up/down movement of tip during etch [24], etchant composition and temperature [25], etching inside an etchant permeable or impermeable plastic tube (fiber jacket) for different etchant concentrations [26], variation of etchant concentration during etch [27], and meniscus around fiber at interface between HF etchant and oil layer of varying density together with subsequent heating of the tip [28].

However, for POFs, only linear tapering has been demonstrated so far with the chemical etching method [29,30,31,32]. In the first paper on this by Merchant et al. [29], etching was made within 1 h to several days with acetone-based solvent mixtures on declad POFs poly(methyl methacrylate) (PMMA) core sections and not at fiber tips. To reduce induced cracks and crazes due to the primary etchant acetone, it was mixed with methyl isobutyl ketone (MIBK) and distilled water. In other studies, POF etching has often been made in solvent mixtures of acetone and methanol [31,32]. We here demonstrate, for the first time, a controllable and simple chemical etching method for CPC tip fabrication on POFs, which can be used for batch production. We further modelled the etching process and characterized the manufactured CPC fiber tips in a fluorescence-based glucose dummy sensor for its improvement in *η*, which was found to be 3.16.

## 2. Materials and Methods

The fiber used to manufacture the CPC tip was a Mitsubishi ESKA^®^ step index multimode fiber, purchased from Edmund Optics^®^. The specifications according to Edmund Optics^®^_,_ found important for CPC formation are shown in Table 1.

**Figure 1:**
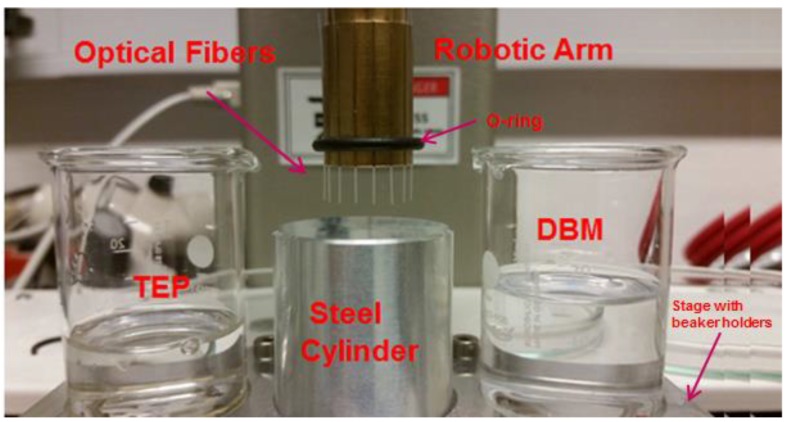
Setup for chemical etching of the fibers for compound parabolic concentrator (CPC) tip formation. The tips are first immersed in dibromomethane (DBM) for etch of the core, then cleaned with distilled water and finally immersed in triethylphosphate (TEP) for etch removal of the cladding.

The fluorine polymer cladding was found to be (C_2_F_2_H_2_)_n_, or Poly(vinylidene difluoride), (PVDF) by Attenuated Total Reflection (ATR) in earlier work on bonding membranes to this kind of POFs [33]. PVDF is a thermoplastic chemically inert non-stick polymer material exhibiting unusual piezoelectric, ferroelectric, and pyroelectric properties. The ideal parameters for a CPC tip on this fiber, which were found in our previous work [16], are an input aperture diameter of 240 µm, an output aperture diameter of 82 µm, and a length of 445 µm. The CPC tip with these specifications will ideally couple all the light collected into the acceptance angle of the straight part of the fiber for interrogation, as demonstrated by Zemax modelling. To make a CPC fiber tip with such specifications, we used chemical etching. To have fibers with equal low residual stresses, all fibers were annealed at 90 °C for 1 day before etching. Furthermore, all fibers were end face polished to the same surface quality by the company FiberFin, Inc. (Yorkville, IL, USA). A setup was developed to chemically etch several fibers simultaneously to make a batch of CPC fiber tips. The setup consisted of a cylindrical fiber holder with several grooves equally distanced from each other, as shown in Figure 1.

In each groove, a fiber of 35 mm length was placed and fixed with the help of an O-ring. The advantage of using an O-ring fixture, is that it allows sliding the fibers inside the grooves to adjust their vertical position. The fiber holder was attached to the robotic arm, which moved in the z-direction to immerse the fibers into the solvents for etching. Another part of the setup consisted of a stage with three beaker holders. Two were used to hold the beakers with solvents used to etch and remove the core and the cladding. The middle holder was used for a steel cylinder, which has a smooth surface. The purpose of this steel cylinder was to adjust the vertical position of all the fibers against the smooth surface to avoid any variation in the final CPC tip lengths after etching. Once all the fibers were adjusted to the same level, the robotic arm was programmed to immerse them in the solvents for etching.

**Figure 2:**
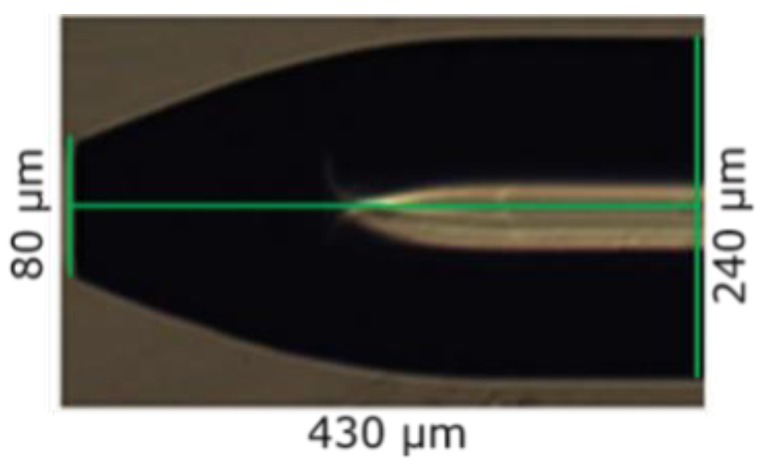
Microscope picture of etched CPC fiber tip with its specific dimensions. The bright zone from the right side of the picture to the center is a reflection from the microscope light source.

The formation of the CPC fiber tip has 3 main steps: (1) Etching of the fiber core, (2) removal of the fiber cladding from the CPC tip, and (3) cleaning of the fiber tips. Without removing the cladding, fibers were immersed into the beaker containing 99% dibromomethane (DBM) from Sigma-Aldrich Denmark A/S (Copenhagen, Denmark), a solvent for etching the PMMA fiber core without etching the PVDF cladding. The immersion depth of the fibers was controlled using the robotic arm to be around 700 µm. The fibers were then left for etching for about 15 min. in dibromomethane (DBM). After 15 min, the fibers were removed from the DBM, cleaned first with dry lint free tissue and then with a tissue with distilled water and immersed in 99.8% triethylphosphate (TEP) from Sigma-Aldrich Denmark A/S (Copenhagen, Denmark), a solvent for etching the PVDF [34] cladding without etching the PMMA core, for 5 min to remove the cladding. In the next step, the fibers were removed from the TEP solution and cleaned with distilled water to remove any residual solvents. A CPC formed by this method, is shown in Figure 2. It had a length of 430 µm and an output diameter of 80 µm, which is very close to the ideal CPC specifications.

## 3. Results and Discussion

### 3.1. Etch Mechanism

Tapering of glass-based fibers by etching usually takes place in the very toxic etchant hydrofluoric acid, HF. In this chemical etch process covalent chemical bonds are broken and small molecules are liberated. Etching of POFs, which are made of thermoplastic polymers, is fundamentally different, because etching takes place by dissolution in a solvent where only long-range cohesive bonding between the polymer chains is broken, i.e., chemically unchanged polymer molecules are separated. Generally, the etch processes in the two kinds of materials are also completely different due to very large differences in etchant diffusivity and solubility. Because of these differences, etching of glass fibers inside an etchant (HF) permeable polymer tube leads to simple fiber diameter reduction [26], also sometimes called step etch or linear etch, in contrast to our observations on POFs with a solvent etchant penetrating the polymer cladding.

We used the theory of Hansen Solubility Parameters (HSP) [35] to select the solvents for POF etching. According to this theory, the cohesive bonding energy density in polymers and solvents can be divided in dispersive, polar, and hydrogen bonding parameter contributions. The dimensionless Relative cohesive Energy density Difference (RED) for a polymer-solvent system is a measure of how well each kind of solubility parameter in a polymer and solvent match each other. For RED < 1, the polymer dissolves in the solvent, if RED = 1 the polymer swells, if RED > 1 there is no attack on the polymer by the solvent. RED numbers depend strongly on specific polymer grades and treatment history, which in most cases makes testing mandatory for reliable solubility predictions. Here, as a guide, we used the polymer and solvent HSP data contained in the Hansen Solubility Parameters in Practice (HSPiP) software version 5.0.06.

Many solvents can etch PMMA. Acetone is most commonly used to etch PMMA based POFs [29,30,31,32]. However, sometimes this solvent leads to cracks and crazes in PMMA POFs [29,32] (RED = 1.58 using PMMA polymer number 66 in the HSPiP polymer database). This does not happen for DBM with RED = 1.05. DBM is also not etching the PVDF cladding, as RED = 1.92. PVDF is very inert and, so far, three solvents capable to etch it have been identified [34], Heaxamethylphosphoramide (HMPA), triethylphosphate (TEP), and dimethylsulfoxide (DMSO), in order of decreasing toxicity. The corresponding PVDF−solvent RED numbers are 0.85, 0.34, 1.28 respectively. For DMSO etch of PVDF we found that heating to about 60 °C was necessary. The corresponding PMMA−solvent RED numbers are 0.41, 1.08, 1.62. Hence, we chose to use TEP because no attack on PMMA during PVDF removal is a requirement and we did not want to complicate the processing by using DMSO and heating.

**Figure 3:**
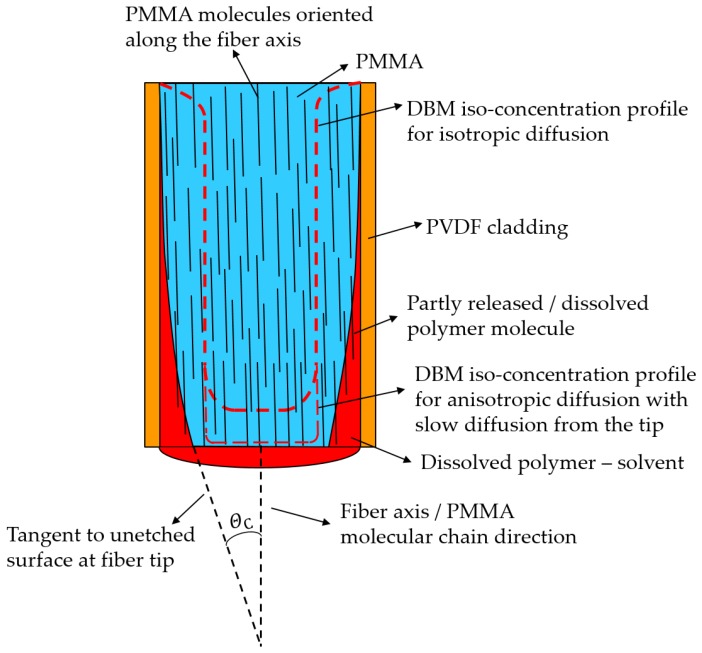
Sketch illustrating the proposed etch mechanism. Only the dipped section of the fiber is shown. Note that the etched fiber tip shape is not following a DBM iso-concentration profile. This is due to PMMA molecular orientation along the fiber axis originating from the fiber drawing process determining local etch speed and combined cladding and dissolved polymer buoyancy restriction of polymer transport away from the fiber.

We believe the POFCPC formation is governed by the combined effect of the following six shaping mechanisms or factors, see Figure 3:

1. The concentration profile of anisotropically in-diffused solvent at the fiber tip region. From the fiber drawing polymer molecules are oriented along the fiber axis [36] leading to crystallinity. Diffusion cannot be expected to be isotropic in an anisotropic polymer material [37,38,39]. Thus, it could be that DBM diffusion from the fiber tip along the fiber axis-oriented molecules is much slower than perpendicular to the molecular chain direction. This provides a good explanation for both the almost constant fiber length and the sharp edge maintained at the fiber tip at all etch times.
2. The cladding acts as a membrane limiting the DBM transport to the PMMA fiber core. If it is assumed that the DBM partition coefficient at the two PVDF interfaces is 1, the DBM flux *F* though the membrane is $F=\frac{D}{L}\cdot S=P\cdot S$, where *D* is the DBM diffusivity in PVDF, *L* is the PVDF thickness, *S* is the solubility of DBM in PVDF, and *P* is the DBM permeability of PVDF [38]. This limitation of the DBM access to the core will, to some extent, slow down etch from the side of the fiber.
3. The DBM swells the PMMA before dissolution [35,40] and swelling strain $\varepsilon_{S}=\beta C$, where β is the swelling coefficient and *C* the DBM concentration [41], is introduced in the material.
4. The cladding acts as semi-permeable membrane: It is permeable towards DBM but impermeable for the much larger released PMMA polymer chains. This is unlike glass fiber etch inside HF permeable polymer tubes [26] where some transport of the small etch product molecules silicon dioxide (SiO_2_) and hexafluorosilicic acid (H_2_SiF_6_) out through the tube is possible. Thus, in our case, the cladding restricted the polymer swelling and escape of dissolved polymer into the solvent. Furthermore, the polymer-solvent mixture will have a lower density than the surrounding pure DBM (density: 2.5 g/cm^3^) and thus, due to buoyancy, dissolved material will tend to stay at the fiber tip and in the region between cladding and un-dissolved PMMA core. This is also unlike glass fiber etch inside HF permeable polymer tubes where the product mixture will have higher density than the surrounding HF (density: 1.15 g/cm^3^). Transport of core polymer out of the PVDF tubing during etch is explained by the osmotic pressure exerted by the polymer solute winning over the buoyancy pressure. The contribution to the osmotic pressure is largest far away from the tip where the concentration of polymer solute in DBM is highest. High polymer concentration at the same time decreases etch speed. At the fiber tip, DBM has direct access to the core, which leads to lower osmotic pressure contribution and higher etch rate.
5. Anisotropic etching only due to molecular orientation and length. The crystallinity itself leads to higher etch speed from the vertical side compared to etching from the tip, where the oriented molecule chains are locked deeper into the polymer matrix. At a certain distance from the tip, the etch speed is determined by the angle Θ between the tangent to the etched surface and the fiber axis/molecular orientation, see Figure 3. The higher the angle, the deeper the solvent has to diffuse to free a long molecule. Due to this, the iso-concentration profile corresponding to etch far from the tip might be deviated from close to the tip and lead to the parabolic shape. Note here, that at the fiber tip, there seems to be a critical angle $\Theta_{C}=23{}^{\circ}$ beyond which etching almost stops. This behavior might have similarities to anisotropic etching along crystal planes observed in wet potassium hydroxide (KOH) etching of single crystalline silicon wafers where for instance etching in the <100> direction is 400 times faster than in the <111> direction [42,43]. This results in a sharp edge at the tip, no matter the etch time, which would not have been seen for an isotropic material. For an isotropic polymer etch, edges are rounded off according to the rounded off diffusion profile.
6. Anisotropic residual stresses. In the fiber, even after annealing, there will be some residual stresses along the fiber, which are zero at the free plane-cut tip surface. Stresses lower activation energies for diffusion and etch and hence this enhances effects one and five.

**Figure 4:**
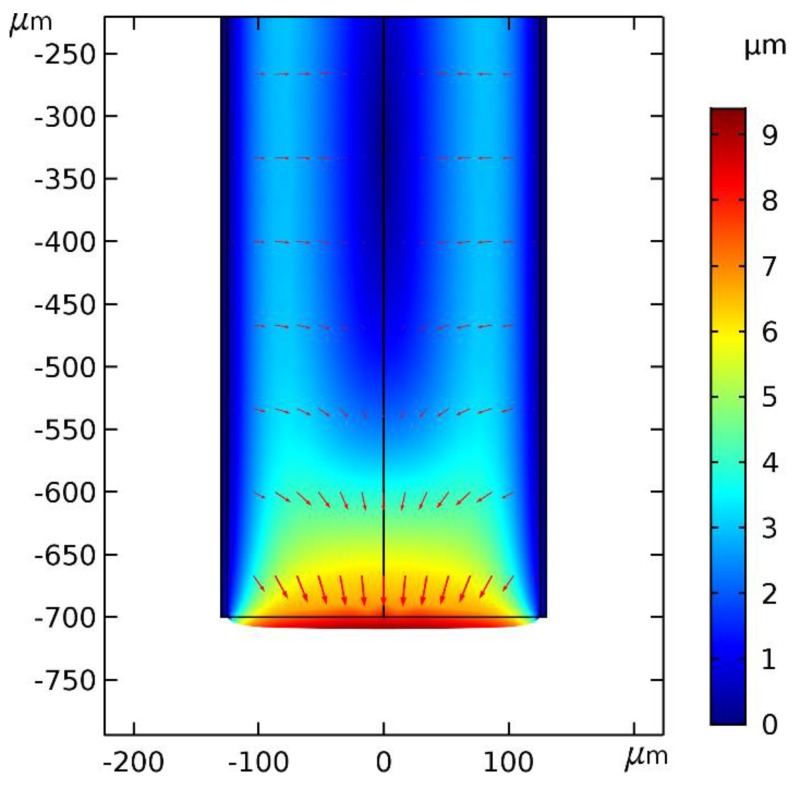
COMSOL Multiphysics 5.3a simulation of PMMA total displacement (surface) and displacement field (arrow surface) after 100 s for the POF dipped 700 µm into DBM. Involved physics in the simulation: Transport of diluted Species, Solid Mechanics and Hygroscopic Swelling. Swelling is assumed isotropic. Dissolution (free PMMA molecules) is not included in the simulation. We assume no movement of the cladding. Real values for DBM diffusion constants, hygroscopic swelling coefficient, solubility in Poly(vinylidene difluoride) (PVDF), and PMMA are not known, but also not important for illustration of the combined mechanism. However, in the shown simulation, diffusion constants for DBM was assumed to be $5\cdot 10^{-12}$ m^2^/s in PMMA perpendicular to the fiber axis and in the PVDF cladding. Along the fiber in PMMA it was assumed to be $5\cdot 10^{-14}$ m^2^/s. DBM concentration at all surfaces was at all times set to be equal to some arbitrary solubility of DBM in PVDF. From the simulated displacement field, it is seen, that PMMA was locally moved by swelling in directions and extents, which could very well lead to the CPC shape. This picture is unchanged if e.g., the diffusion is assumed to be isotropic and/or the cladding is thicker.

**Figure 5:**
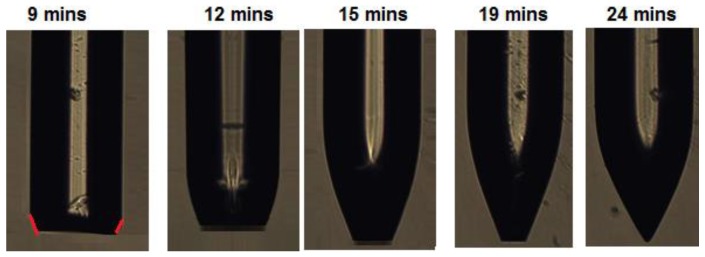
Formation of CPC fiber tip. Five fibers were used. Each fiber was etched with cladding using different etching times. To make the shape visible, the cladding was removed in each case before taking the picture. The picture at 9 min is marked with red tangent lines to indicate the angle $\Theta_{C}$ with the dominant PMMA molecular direction along the fiber axis. The vertical bright zones on the fibers are reflections from the microscope light source.

Strong anisotropic removal of PMMA was evidenced in two ways. First, retraction of the core tip from the original cut plane could not be seen as depicted in Figure 3. However, such fiber tip retraction is observed in glass fiber etch inside HF permeable polymer tubes, indicating more isotropic glass removal [26]. Secondly, it was confirmed by immersing a 1 mm thick declad PMMA core fiber in DBM and observing the thickness and length changes. COMSOL Multiphysics simulations, see Figure 4, show that alone 1, 3, and 4 from above, i.e., anisotropic DBM diffusion into the PMMA fiber core from the tip and through the semi-permeable PVDF cladding and the induced PMMA swelling displacement field, can account for much of the observed shaping. Thus, the displacement field in Figure 4 shows that PMMA was locally moved inward and downward by swelling. In sufficiently swollen regions, this movement may loosen the PMMA along the arrow directions and in this way control the etch/dissolution leading to the CPC shape. Note that, at a distance of approximately 400 µm from the tip, the movement was, at all times, only small and inward, which corresponds well with the observed CPC length, see Figure 2. This demonstrates insensitivity of CPC length on dipping depth beyond approximately 400 µm and buoyancy of dissolved material. Figure 5 shows fiber tip shapes obtained after different times and after removal of the cladding to make the shape more visible. The central picture after 15 min is the best CPC shape also shown in Figure 2. From the pictures, $\Theta_{C}$ has been found to be approximately 23° for the first four etch times from the left. After 24 min etch there was no longer a plane-cut surface at the fiber tip and now $\Theta_{C}$ at the fiber tip had changed to approximately 30°.

### 3.2. Characterization

**Figure 6:**
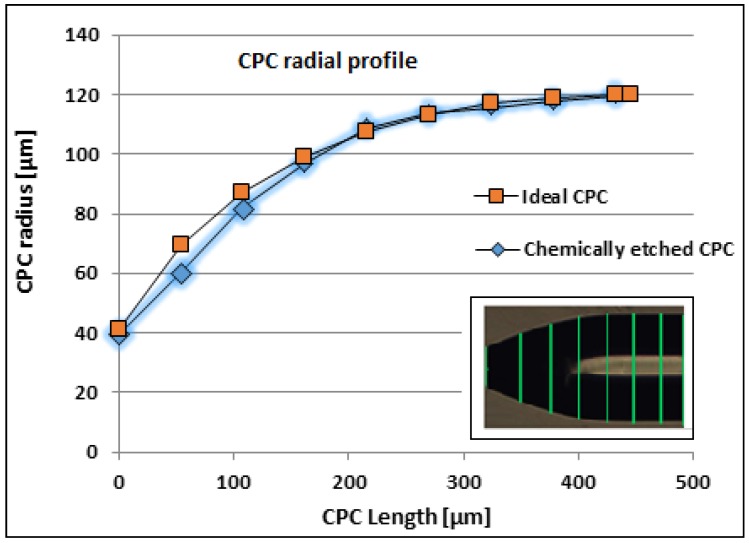
Comparison of ideal and chemically etched CPC profile. The CPC length values are from the fiber tip of one of the eight CPCs. The microscope measurement precision was ±1 µm.

A batch of eight CPC fiber tips was made using the chemical etching method. To characterize them geometrically, we reconstructed the chemically etched CPC profile by measuring the coordinates from the CPC picture radius at different positions, as shown in the inset of Figure 6 and plotted in Figure 6 together with the ideal CPC profile. The figure shows that the chemically etched CPC shape was close to the ideal CPC shape, even though the last part of the CPC tip was more linear and a little too much material was etched away here. This can be changed by adjustment of the mentioned shaping mechanisms. Thus, a method to avoid the over-etch could be by preserving more of the polymer molecular orientation from the drawing process e.g., reducing the degree of annealing relaxation in the pretreatment of the fibers. In the PMMA core, this would result in higher anisotropy of DBM diffusivity and residual stress, together with a higher degree of molecule chains locked deeper into the polymer matrix close to the tip, cf. the etch mechanisms one, five, and six described above, and possibly a lower etch speed in this region. However, this could at the same be at the expense of over-etch farther away from the tip and instead getting a tip shape deviating more from the CPC shape here. Nevertheless, this shows that the chemical method is an effective method to make high quality CPC fiber tips close to the ideal shape.

The fluorescence coupling efficiency of the CPC tip has been characterized using a glucose sensor chemistry used and explained in detail in our previous work [16]. The assay chemistry consists of a glucose binding protein labelled with “assay” fluorophore (Alexa fluor 594: λ excitation = 590 nm, λ emission = 618 nm) and a glucose analog, which is labelled with the dye hexamethoxy crystal violet HMCV1. In absence of glucose molecules, they make a FRET (Foster Resonance Energy Transfer) pair [16], which quench the emitted fluorescence intensity of the “assay” fluorophore. In presence of glucose molecules, they compete with the glucose analog molecules to attach to the protein, thus breaking the FRET pair. As a result, the fluorescence quenching is reduced and assay fluorophores retain their initial fluorescence intensity. Therefore, the glucose concentration in the chemistry is correlated to the detected fluorescence intensity.

**Figure 7:**
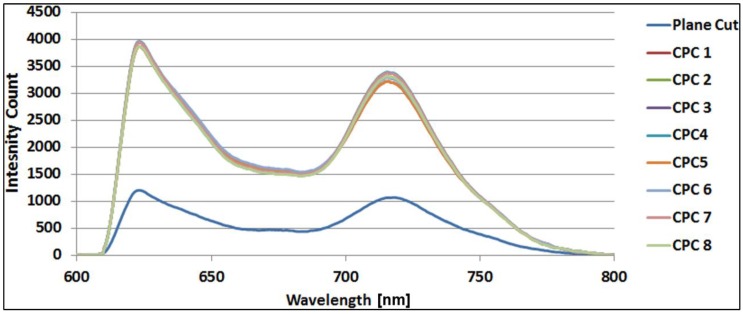
Spectrum for plane-cut and CPC fiber tips for dummy sensor.

The sensor chemistry also contains a reference fluorophore (Alexa fluor 700: λ_excitation_ = 633 nm–647 nm, λ_emission_ = 723 nm), unaffected by the glucose concentration. The purpose of the reference fluorophore is elimination of any unwanted fluctuations in the detected fluorescence intensity, which can either be caused by the fluctuations in the light source used for exciting the assay or the coupling between the assay and the light source. The ratio between the assay and reference fluorescence determines the absolute glucose concentration. The increment factor determining the improvement in coupling efficiency of the CPC tip is defined as the ratio of the detected assay fluorescence intensity of the CPC-tipped sensor and the plane-cut sensor at 618 nm. We determined the increment factor using an optical setup and dummy sensor configuration to detect the fluorescence spectrum of the chemistry [16]. In the optical setup, light from a broad band LED source (HLMP-EL30-MQ000 from BROADCOM Inc., San Jose, CA, USA) with dominant wavelength of 590 nm passed through a 55 nm excitation filter with 560 nm central wavelength and a beam splitter and was coupled to the fiber by a lens to excite the assay chemistry. The fluorescence from the assay chemistry was picked up by the fiber and passed through a beam splitter and a long-pass emission filter with 610 nm cut-off wavelength to finally reach the spectrometer (USB2000+ from Ocean Optics, Largo, FL, USA). The dummy sensor was a 35 mm long CPC tipped fiber inserted approximately 2 mm into a miniature cuvette filled with the sensor chemistry described in the previous two paragraphs and glucose to reduce fluorescence quenching. The 1 cm long cuvette was made of a non-fluorescing transparent 250 µm inner diameter Tygon tube sealed in one end with adhesive and a fiber piece inserted into the tube giving a cuvette compartment length of approximately 4 mm. Thus, the distance between the CPC fiber tips and the seal was in all cases approximately 2 mm. In a real sensor, this cuvette was replaced with a glucose permeable membrane, which is at the same time impermeable to the sensor chemistry. To eliminate any effect of coupling variations and misalignment from the setup, three measurements were taken for each sensor, by each time making new dummy sensors from the same CPC tipped fibers. The detected average spectra from the eight CPC-tipped and plane-cut fiber sensors are shown in Figure 7; each has been averaged over three measurements.

The batch average increment factor of the CPC fiber tips showed an overall *η* of 3.16. The variation in the increment factor was negligible, which showed that the fiber tips are reproducible. An ideal CPC should show a *η* of 3.96. However, the fiber tips were not perfectly CPC shaped, as the tapered length is 430 µm compared to 445 µm for the ideal shape and the last part of the chemical etched CPC fiber tip was more linear, as shown in Figure 6. The deviation from the ideal increment factor achieved by chemically etched CPC fiber tips was only 20%. This is much less than for CPCs made by using the heat-and-pull method, where the deviation is around 43%. However, 20% deviation is still more than from the CPC fiber tip fabricated using fs laser micromachining, which was only 11.6%. Nevertheless, this method produced high-quality CPC fiber tips and is reproducible with a considerable high fluorescence pickup efficiency that can be further optimized to approach the optimum improvement. More importantly, this method is simple and has an advantage that it can be up-scaled to simultaneously make hundreds or more of CPCs of the same geometry. The process is remarkably simple and has tolerance against environmental perturbations, such as small temperature variations and vibrations. High quality and smooth surfaced fiber tips can be produced with this method and the fact that etching time does not influence the smoothness of the fiber by leaving any residues, makes the handling of the process straightforward and easy.

## 4. Conclusions

We demonstrated a simple nonlinear etching method to manufacture POFCPC tips and presented a model, which can be used to control the tip fabrication and create different shapes. Both experiments and modelling show that, etching the fiber without removing the cladding plays an important role in the formation of CPC shaped fiber tips. The manufactured fiber tips experimentally showed an increment factor of 3.16 in glucose sensor fluorescence pickup efficiency compared to the plane-cut fiber tip. The etching method for CPC tip manufacturing is simple and cost-effective. The method is industry friendly as it can be upgraded for mass scale production for high-quality CPC fiber tips.

## Figures and Tables

**Table 1:** Fiber parameters important for chemical etch CPC tip formation.

Core Material	Cladding Material	Fiber Diameter	Core Diameter	Cladding Thickness
PMMA*	Fluorine polymer	250 µm	240 µm	5 µm

* poly(methyl methacrylate) (PMMA).
